# Dooming Phagocyte Responses: Inflammatory Effects of Endogenous Oxidized Phospholipids

**DOI:** 10.3389/fendo.2021.626842

**Published:** 2021-03-15

**Authors:** Marco Di Gioia, Ivan Zanoni

**Affiliations:** ^1^ Division of Immunology, Harvard Medical School, Boston Children’s Hospital, Boston, MA, United States; ^2^ Division of Gastroenterology, Harvard Medical School, Boston Children’s Hospital, Boston, MA, United States

**Keywords:** oxidized phospholipids, oxPAPC, inflammation, immunometabolism, inflammasome, atherosclerosis, lung, COVID-19

## Abstract

Endogenous oxidized phospholipids are produced during tissue stress and are responsible for sustaining inflammatory responses in immune as well as non-immune cells. Their local and systemic production and accumulation is associated with the etiology and progression of several inflammatory diseases, but the molecular mechanisms that underlie the biological activities of these oxidized phospholipids remain elusive. Increasing evidence highlights the ability of these stress mediators to modulate cellular metabolism and pro-inflammatory signaling in phagocytes, such as macrophages and dendritic cells, and to alter the activation and polarization of these cells. Because these immune cells serve a key role in maintaining tissue homeostasis and organ function, understanding how endogenous oxidized lipids reshape phagocyte biology and function is vital for designing clinical tools and interventions for preventing, slowing down, or resolving chronic inflammatory disorders that are driven by phagocyte dysfunction. Here, we discuss the metabolic and signaling processes elicited by endogenous oxidized lipids and outline new hypotheses and models to elucidate the impact of these lipids on phagocytes and inflammation.

## Introduction

Immune cells are strategically distributed in the body and react rapidly to internal and external cues, thereby controlling tissue homeostasis. In particular, phagocytes such as macrophages play a key role not only against pathogen invasions, but also in organ function. Macrophages regulate remodeling and maturation of synapses during brain development (1), as well as bone formation (2), electrical conduction in cardiomyocytes (3), gastrointestinal motility (4) and insulin sensitivity (5), among others. Thus, perturbations in the biology of these cells, or in the quality of their responses, have a profound impact on the etiology and development of several pathologies. Classically, phagocytes respond to stress stimuli, which trigger inflammatory programs and eliminate the source of stress, and/or support adaptation mechanisms. The persistence and accumulation of stress signals may lead to the exacerbation and persistence of inflammation, and thus to tissue dysfunction. Endogenous oxidized phospholipids have been shown to function as stress signals that may profoundly impact the activity of innate immune phagocytes.

The arachidonic acid-containing phospholipid 1-palmitoyl-2-arachidonoyl-sn-glycero-3-phosphocholine (PAPC) is a constituent of the plasma membrane of every cell type (6)), lung surfactant (7–9), and circulating lipoproteins (10). PAPC reacts with oxygen on the *sn-2* chain to create a mixture of oxidized phospholipids, collectedly referred to as “oxPAPC”. Although exogenous acute administration of oxPAPC before the encounter with an inflammatory moiety reduces the subsequent immune response both *in vitro* and *in vivo* (11–13), endogenous production and accumulation of oxPAPC during pathophysiological conditions are strictly associated with the onset of a detrimental chronic inflammation. In fact, oxPAPC accumulates in apoptotic cells (14–16), microparticles released by activated or dying cells (17, 18), oxidized low density lipoproteins (oxLDLs) (19) and oxidized pulmonary surfactant (20). oxPAPC also actively modulates cellular signaling processes, and contributes to the initiation and amplification of inflammation in atherosclerosis (21), lung injury and viral infections (20), non-alcoholic steatohepatitis (NASH) (22), colitis (23), leprosy (24), UV-irradiated skin (25), myocardial and hepatic ischemia (17, 18, 26), multiple sclerosis (27, 28) and inflammatory pain (29, 30).

In this review, after an overview of the capacity of lipids to modify several signaling processes, we focus on the role of *endogenous non-enzymatically oxidized phospholipids* (oxPLs) such as oxPAPC, in sustaining and enhancing inflammatory disorders. In particular, we discuss how oxPLs modulate pro-inflammatory responses in immune cells, with special attention on the crosstalk between metabolic and signaling pathways in phagocytes; we discuss how oxPAPC affects the pathophysiology of inflammatory diseases such as atherosclerosis and lung infections.

## Lipids Modulate Cellular Signaling Processes

Lipids not only serve a structural role in membranes and function as a source of energy, but they are able to modulate cellular signaling processes. This last task is performed *via* several mechanisms, which are not mutually exclusive.

Alteration of the relative abundance of lipid species that constitute the cellular “lipidome” (31) is one of such mechanisms. Changes in the lipid composition of the plasma membrane can modify its mechanical proprieties, such as curvature and fluidity, and can thereby affect several membrane-dependent events, including phagocytosis (32), ion channel gating (33), and signal transduction (34). Local distribution of lipids in intracellular organelles also coordinates their morphology and functionality, as has been described for mitochondria in which the ratio of the phospholipids phosphatidic acid (PA) and cardiolipin (CL) directs fusion or fission dynamics (35, 36). Remodeling of the cellular lipidome may be driven by perturbations of the extracellular milieu, as occurs during atherosclerosis progression, wherein diet-derived lipid deposition affects the lipid content of phagocytes and thus the features of their cellular processes (37). Alternatively, the remodeling can be actively governed by the cell that, by activating a specific set of enzymes, reshapes its lipid pool to trigger an optimal response toward a stress factor. This is the case when immune cells (such as macrophages) modify their lipidome configurations in relation to the nature of stimulus they receive (38). In this manner, the activation of different classes of Toll-like receptor (TLR) induces distinct lipidomes in macrophages that are necessary to promote an appropriate inflammatory response (38–41).

A second mechanism utilized by lipids to modify cellular signaling is the co- and post-translational protein modification, referred to as “lipidation”. Several lipids are covalently attached to proteins and change the folding of the proteins, their half-life, association to membranes and other proteins, sub-cellular localization, and binding affinity to their co-factors and substrates (42). Palmitoylation (the addition of palmitate to a cysteine residue (43)), is one of the best characterized lipid modifications and controls the stability, trafficking and functionality of the target protein. This has been shown for the nucleotide oligomerization domain (NOD)–like receptors 1 and 2 (NOD1/2), which are responsible for detecting bacterial products in immune cells. NOD1/2 require palmitoylation in order to be recruited to bacteria-containing endosomes and to function therein (44). Lipids are also an important source of acetyl-coenzyme A (acetyl-CoA) (45), which is a central metabolite that drives protein acetylation and thereby controls not only gene expression through histone modification, but also other key cellular processes such as DNA repair of double-strand breaks, cell cycle, cellular signaling, protein conformation, autophagy and metabolism (46). For example, acetylation supports the assembly and activation of the NACHT, LRR and PYD domain-containing protein 3 (NLRP3) inflammasome (47), an innate immune sensor that responds to several exogenous and endogenous stressors (48).

Lastly, lipids can be chemically and structurally modified to impact the signaling process. In this case, specific cellular enzymes catalyze definite modifications to a target lipid. Eicosanoids and steroid hormones are lipids that are produced *via* a spatially and temporally controlled multi-step mechanism, in which arachidonic acid (or other related polyunsaturated fatty acids (PUFAs)) and cholesterol, respectively, are converted into their final biological active forms by a succession of enzymatic reactions (49, 50). G protein-coupled receptors for eicosanoids, and nuclear receptors for steroid hormones then coordinate regulatory responses that control cellular as well as systemic metabolism, development, and tissue homeostasis (49, 50). Production of new lipidic molecules can also occur in a non-enzymatic manner: lipids can spontaneously react with free radical species present in both extracellular and intracellular compartments and give rise to a wide variety of biologically active products. PUFAs can undergo uncontrolled nitration (51), sulfation (52) and oxidation (19) during tissue stress conditions. For example, prostaglandins are eicosanoids produced by the strict guide of cyclooxygenase (COX) enzymes, on the contrary, isoprostanes (53) are prostaglandin-like compounds formed by non-enzymatic peroxidation of the same COX’s substrates during oxidative damage. OxPAPC is another important example of a class of chemically modified lipid moieties that are implicated in the development of inflammatory disorders.

## Oxidized Phospholipids Boost and Sustain Inflammation In Phagocytes

oxPLs *per se* are weak inductors of pro-inflammatory cytokine production by phagocytes, and they only slightly upregulate the expression of interleukin-6 (IL-6) and IL-1β (20, 54, 55). Nevertheless, oxPLs potently boost and extend the inflammatory capacity of dendritic cells (DCs) and macrophages (56–60). In particular, prolonged exposure of phagocytes to oxPLs strongly potentiates the production of pro-inflammatory cytokines thanks to the ability of oxPLs to reprogram the mitochondrial metabolism of the phagocytes (60) and to activate the release of IL-1β, while maintaining cell viability (56).

### Metabolic Activities of Oxidized Phospholipids in Phagocytes

Depending on the type of signal that is detected, phagocytes reprogram their cellular metabolism differently, in order to support a proper response (61). The Gram-negative bacteria lipopolysaccharide (LPS), one of the best characterized exogenous stressors, induces global rewiring of the major metabolic pathways that dictate microbial killing processes, production of pro-inflammatory mediators and the control of cell viability (62–66). LPS-activated phagocytes increase glycolysis and the pentose phosphate pathway (PPP), which in turn provide ATP and metabolic intermediates that support protein translation and the biosynthesis of several macromolecules, such as the fatty acids, necessary for the expansion of secretory compartments (63, 65, 67–70). In the LPS-activated phagocytes, mitochondrial activity undergoes several alterations: i) the tricarboxylic acid (TCA) cycle is “broken” in two places, due to a reduction in isocitrate dehydrogenase (IDH) expression and a decline in succinate dehydrogenase (SDH) functionality; and ii) the electron transport chain (ETC) is suppressed, mainly due to the production of nitric oxide (NO) (63, 64, 66, 71). These changes shorten the cell’s lifespan (66) and allows the accumulation of key metabolites such as citrate, succinate and itaconate, which control the activity of transcription factors and effector molecules such as hypoxia-inducible factor 1-alpha (HIF-1α) (63) and the NLRP3 inflammasome (72).

**Figure 1:**
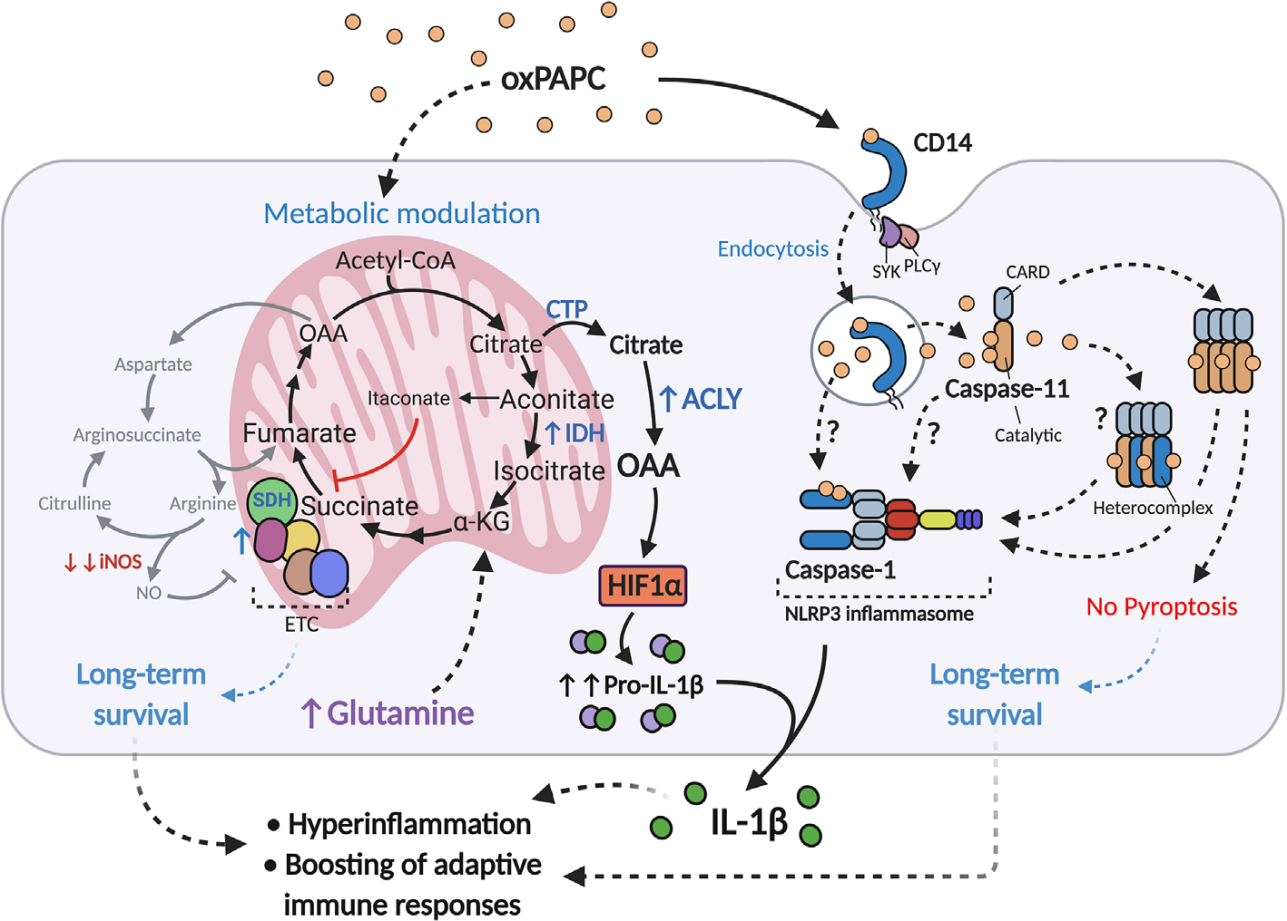
oxPAPC boosts inflammatory responses in LPS-activated macrophages. Upon LPS encounter and/or during atherosclerosis development, oxPAPC induces a metabolic remodeling state in phagocytes, termed hypermetabolism, that is characterized by 1) boosting of mitochondrial activity *via* iNOS inhibition and ETC protection; 2) sustaining the TCA cycle with glutamine and upregulation of IDH; and 3) upregulating ACLY. These events result in the conversion of citrate to OAA, which in turn stabilizes HIF-1α and increases production of pro-IL-1β. OxPAPC is also transported into the cytosol *via* the endocytic module CD14-SYK-PLCγ, where it interacts with caspse-11/4 and induces oligomerization of this enzyme. oxPAPC may also interact with caspase-1, to form caspase-11/4/5-1 hetero-complexes, or to activate the NLRP3 inflammasome. These processes, termed hyperactivation, lead to IL-1β cleavage and release, but not to pyroptosis.

Recent evidence suggests that oxPLs can modify the metabolism of phagocytes, as reported for adipose tissue macrophages (ATM) in obese animals (73) and for circulating and tissue-resident monocytes/macrophages in atherosclerotic mice (60). Prolonged exposure of LPS-activated macrophages to oxPAPC (referred to hereafter as LPS+oxPAPC) profoundly interferes with the behavior of the mitochondria, and induces a novel metabolic state, termed hypermetabolism, that enhances the production of pro-inflammatory cytokines (60) (**Figure 1**). Mitochondrial activity is potentiated in cells treated with LPS+oxPAPC, sustaining the TCA cycle and respiration. The expression of IDH is selectively increased, and NO production is severely impaired, thus preventing the loss and dysfunction of ETC complexes. In this manner, the intact TCA cycle leads to the export of citrate into the cytosol, where it is converted into acetyl-coA and oxaloacetate (OAA) by the enzyme ATP-citrate lyase (ACLY). In turn, OAA, probably through direct inhibition of prolyl hydroxylases (PDH) (63, 74), stimulates stabilization of HIF-1α, which potently increases the transcription and production of IL-1β. This entire process is fed by glutamine catabolism rather than by glycolysis, even though LPS+oxPAPC cells continue to conserve a high rate of glucose utilization, as occurs in response to LPS only. Notably, glutaminolysis also plays a key role in epigenetic reprogramming, which controls long-term macrophage responses such as their inflammatory polarization and trained immunity (75–77). This mechanism is further reinforced by acetyl-coA, formed by ACLY, which directly supports histone modifications and thereby facilitates the transcription of target genes (78–80). In addition, oxPAPC treatment is sufficient to potently increase the mitochondrial potential (Δψ_m_) of phagocytes (60), which is the gradient of the electric potential on the inner mitochondrial membrane generated by ETC proton pumps (81). Δψ_m_ has been implicated in several cellular processes in addition to ATP synthesis: these include production of reactive oxygen species (ROS), cell proliferation, functionality of sirtuin deacetylases, cell renewal, and transcription factor activity (82–85). Thus, the conserved and increased mitochondrial fitness induced by oxPLs, possibly assisted also by production of a redox-balancing response (86), may prolong the lifespan of macrophages, as has been described in atheromas (87) and lung injuries (88) - and sustain their inflammatory signature. We propose that all of the metabolic effects induced by oxPLs work in concert, favoring the persistence of long-lived, detrimental, pro-inflammatory phagocytes and collectively contributing to the development of chronic inflammatory diseases.

### Inflammasome Activation by Oxidized Phospholipids

Phagocytes are equipped with receptors that allow them to respond to stress stimuli. In particular, inflammasomes are multiprotein platforms that comprise a sensor protein (i.e. NLRP3), inflammatory caspases (i.e. caspase-1) and an adapter protein (i.e. apoptosis-associated speck-like protein containing a caspase recruitment domain (CARD) – ASC); together, inflammasomes integrate various *non-self* and *self*-signals and induce the secretion of active IL-1β and IL-18 (89). Activation of inflammasomes involves two steps: i) a priming step, generally induced by exogenous molecules *via* TLRs (e.g., LPS and TLR4), that is necessary for the expression of pro-IL-1β (an inactive form of IL-1β) and inflammasome components; and ii) an activation step, whereby a repertoire of intracellular stimuli lead to inflammasome assembly and enzymatic activation of dedicated caspases, resulting in the processing and release of IL-1β through a lytic cell death program (pyroptosis). Typically, perturbations in homeostasis of the cytosolic compartment, such as organelle dysfunction (90–93), ROS production (94, 95), ion flux (96–98), and metabolic alterations (99), prompt “canonical” activation of the NLRP3 inflammasome, while direct recognition of intracellular LPS by caspase-11/4/5 triggers “noncanonical” activation of the inflammasome (100–102). In this latter pathway, LPS elicits oligomerization of caspase-11/4/5, and its activation by auto-proteolytic cleavage (103); this then induces plasma membrane pore formation *via* gasdermin D (GSDMD) (104, 105) and subsequent potassium efflux (106) that, in turn, causes NLRP3 inflammasome activation, pyroptosis and IL-1β secretion.

Extracellular oxPLs can reach the cytosol *via* plasma membrane receptors such as scavenger receptors (107). As with LPS (108), oxPAPC is also a cargo for CD14 (57), which induces internalization of the oxPAPC, and triggers an endocytic process that is mediated by phospholipase C γ (PLCγ) and spleen tyrosine kinase (SYK). How oxPAPC leaves the endosome and enters the cytosol is a mystery. We suggest that other oxPL-specific receptors, such as Transmembrane Protein 30A (TMEM30A) (109) mediate this relocation, but we cannot rule out the possibility that the oxPAPC itself alters the composition of the endosomal membrane and provokes its own leakage from intracellular organelles into cytosol (110). Additionally, oxPLs can be produced intracellularly in response to cellular stress. For example, a recent report showed in a model of age-related macular degeneration that retinal pigmented epithelium cells produce oxPAPC, which supports their pro-inflammatory activity and their role in the development of pathology (111).

Once in the cytosol, oxPAPC binds caspase-11/4/5 and triggers an atypical inflammasome activation, culminating in active release of IL-1β, in the absence of pyroptosis (56) (**Figure 1**). This process, called “hyperactivation”, is critical not only for establishing local long-term inflammation, but also for promoting a strong adaptive immune response (56, 112). The persistence of IL-1β-producing DCs in lymph nodes or in the aortic wall (113), can boost T cell activation, proliferation, and Th1/Th17 polarization, thereby further sustaining local and systemic chronic inflammation.

Inflammasome activation governed by hyperactivation differs from non-canonical inflammasome activation driven by LPS. In fact, LPS and oxPAPC are believed to interact with different domains of caspase-11/4/5, and differentially modulate the downstream effects of this enzyme (56). The highly hydrophobic lipid A moiety of LPS binds the CARD domain of caspase-11/4/5, where basic residues are required for interaction with the phosphate head groups of lipid A (102). Upon engaging LPS, caspase-11/4/5 undergoes oligomerization and activation. However, the exact nature of interactions between oxPAPC and caspase-11/4/5 are still debated (56, 114). The first study on oxPAPC-caspase-11/4/5 of Zanoni et al. using surface plasmon resonance and pull-down approaches, reported that oxPAPC binds the catalytic domain of caspase-11/4/5, and not its CARD domain (56), which enables oxPAPC to promote caspase-11/4/5 oligomerization but does not trigger its enzymatic activity (56). Later on, Chu et al. confirmed the interaction between oxPAPC and caspase-11/4/5, but they found that oxPAPC competes with LPS for the CARD domain of caspase-11/4/5, thus preventing downstream LPS-initiated signaling (114). Although more experiments will be needed to unveil the complex nature of the interactions between oxPAPC, LPS and caspases, a possible explanation for the discrepancies described in the previous studies is that individual oxPAPC constituents bind caspase-11/4/5 in diverse positions, with different affinity and *via* more than one mechanism. In particular, oxPAPC’s interaction with proteins occurs *via* at least two mechanisms. Electrophilic oxPAPC components such as 1-palmitoyl-2-(5, 6-epoxyisoprostane E2)-sn-glycero-3-phsphocholine (PEIPC) covalently bind cysteine residues and modulate the activity of their protein targets. This type of interaction has been previously established for H-Ras (115), transient receptor potential cation channel, subfamily A, member 1 (TRPA1) (30), and for Kelch-like ECH-associated protein 1 (Keap-1) (116). Of note, no cysteine residues are present in the CARD domain of murine as well as human caspase-11/4/5 (117), but such residues are relatively abundant in its catalytic subunit. These data support the observation that oxPAPC selectively interacts with the catalytic portion of caspase-11/4/5 rather than competing with LPS for binding to the CARD domain (56). Alternatively, oxPAPC components that incorporate a terminal γ-hydroxy (or oxo)-α,β-unsaturated carbonyl in their *sn-2* chain interact with proteins *via* electrostatic interactions. For example, positively charged residues in the scavenger receptor CD36 are necessary for interactions of the receptor with 1-palmitoyl-2-(5-keto-6-octene-dioyl)-sn-glycero-3-phosphocholine (KOdiAPC) (118, 119). These interactions mirror LPS binding mechanisms identified for LPS binding protein (LPB) (120), caspase-11/4/5 (102), and the newly discovered intracellular LPS receptor guanylate-binding protein 1 (GBP1) (121), which have also been implicated in the interaction of oxPAPC with caspase-11/4/5 (114).

The oligomerization of caspase-11/4/5 induced by oxPAPC is sufficient to stimulate the NLRP3 inflammasome, even in absence of its catalytic activity. Potassium efflux, a downstream effect of caspase-11/4/5 activation, is not required for IL-1β release from oxPAPC-treated DCs (56), which suggests that “silent” caspase-11/4/5 aggregates can also work in other ways to activate NLRP3 inflammasome.

oxPAPC also directly binds caspase-1 (56), as was identified in RAW 264.7 macrophages with use of tandem mass spectrometry (122). We postulate that the hetero-complexes are composed of caspase-11/4/5 and caspase-1, in which the lack of caspase-11/4/5 activity is balanced by the activity of caspase-1. Also, that engagement of caspase-1 by oxPAPC can bypass the requirement for caspase-11/4/5 to start or sustain inflammasome activation. Indeed, after oxPAPC administration, primed DCs that are caspase-11-deficient can decrease - but not abolish - levels of IL-1β, while those that are caspase-1-deficient completely lose the ability to secrete IL-1β (57). Based on this finding, we hypothesize that the oxPAPC-caspase-1 complex can stimulate NLRP3 assembly and activation. However, we cannot exclude the possibility that certain oxPAPC components, depending on their concentration and the responding cell type, can trigger NLRP3 activation also in “canonical mode” (58), through ROS production (58) or metabolic alterations (58, 60).

Once activated by oxPAPC, neither caspase-11/4/5 nor the NLRP3 inflammasome provoke pyroptosis, but the cell nonetheless acquires the ability to secrete IL-1β. How this cytokine is secreted from living cells is unclear, although GSDMD pores are reportedly implicated in this process (59). The pores form small channels for the secretion of cytosolic cytokines, but the lack of a secondary stimulus, such as potassium efflux (see above), may dampen the lytic death program (56, 59). The cell may also activate a repair mechanism that recruits the endosomal sorting complex required for transport (ESCRT) machinery to the site of membrane damage, and eliminate GSDMD pores from the plasma membrane in the form of ectosomes (121). The rapid turnover of the GSDMD pores allows IL-1β secretion but prevents them from causing extensive plasma membrane damage, which thereby protects the cell from pyroptosis. The effects of oxPAPC on mitochondrial activity (see previous paragraph) may also interfere with the mitochondrial damage that is induced by gasdermins (123), and thus may protect the cell from death. Moreover, oxPAPC-potentiated mitochondrial metabolism can lead to accumulation of specific metabolic intermediates that can alter GSDMD functionality. For example, fumarate reacts with GSDMD at critical cysteine residues to form S-(2-succinyl)-cysteine, thwarting its capacity to induce cell death (124). As discussed above for caspase-11/4/5 binding, we speculate that oxPAPC also directly interacts with GS-DMD *via* thiol groups, thus mimicking the effect of cysteine-modifying drugs such as disulfiram, which block GSDMD pore formation (125).

Lastly, fatty acid epoxycyclopentenone, a *sn-2* moiety identified in some oxPAPC components, induces caspase-8 activation and IL-1β secretion (116). Caspase-8 has emerged as a new player in inflammasome induction (89): it participates in an alternative inflammasome activation pathway in human monocytes, wherein TLR engagement is sufficient to trigger inflammasome activation and IL-1β release, without pyroptosis (126). Of note, murine macrophages exposed to oxPAPC for a long time also acquire this capacity after they are stimulated by LPS only - the cells rapidly secrete high amounts of IL-1β, but preserve their viability (60). This phenotype is largely regulated by the metabolism remodeling induced by oxPAPC that boosts mitochondrial activity and favors the accumulation of metabolites; this, in turn, controls transcriptional and epigenetic programs (see previous paragraph). Nevertheless, oxPAPC could also alter the signaling hub mediated by caspase-8, enhance LPS-dependent responses and reshape NLRP3 activity. Thus, although further work is needed to understand whether or not oxPAPC interacts with human and murine caspase-8, and how it does so (directly or indirectly), oxPLs emerge as possible pleiotropic modulators also of alternative inflammasome pathways in both murine and human phagocytes.

## Atherosclerosis: Roles of Oxidized Phospholipid-Activated Phagocytes

Atherosclerosis leads to a chronic and progressive deposition of fatty and fibrous material in arterial walls. This inflammatory condition can lead to a number of serious pathologies known collectively as cardiovascular diseases (CVDs) – these include coronary heart disease, hypertension and stroke (127). Circulating LDLs that accumulate in the intima layer of blood vessels and undergo oxidative modifications are the main initiators of atherosclerosis. However, other stressors may also contribute to this process. For instance, subclinical endotoxemia, which results from gut mucosal leakages induced during chronic infections, obesity, and ageing, may sustain the development of atherosclerosis (128, 129). oxLDLs start an enduring inflammatory reaction that involves multiple cell types, including endothelial cells, smooth muscle cells, resident macrophages and monocytes (127). In particular, activated macrophages proliferate locally (87, 130), and later, monocytes recruited from bloodstream sustain plaque formation (130). These phagocytes produce inflammatory mediators, and favor accumulation of lipid and lipid-laden cells called foam cells. Foam cells originate from macrophages as well as monocytes (130), and by metaplasia of smooth muscle cells (131), gather and progressively form a lipid-rich necrotic core, which increases over time. Non-immune cells also contribute to inflammation and deposition of extracellular material and promote plaque instability and rupture, with severe risk of thrombosis or other complications (132).

**Figure 2:**
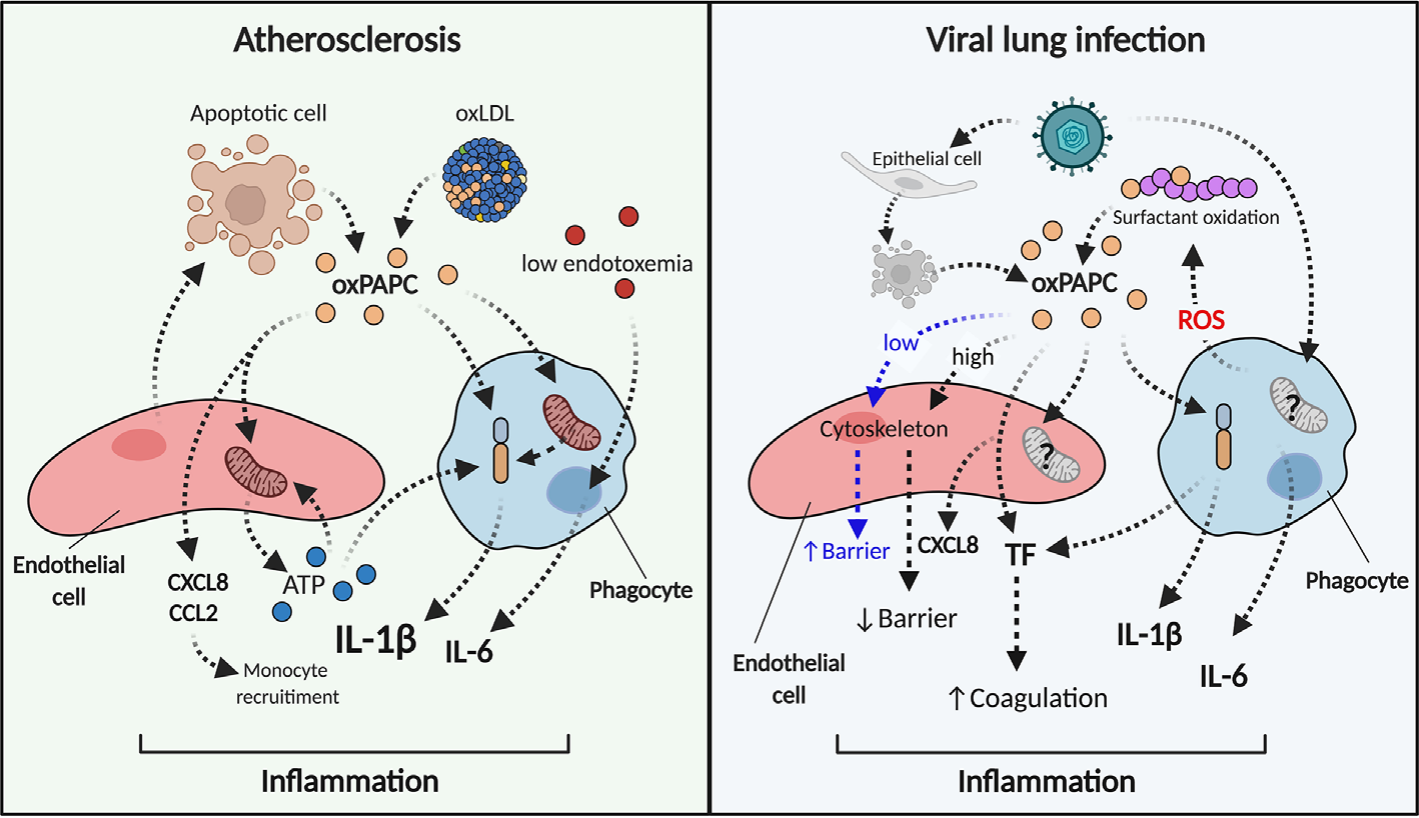
oxPAPC triggers and sustains inflammation in atherosclerosis and viral lung infections. During atherosclerosis (left) oxPAPC released from dying cells or contained in oxLDL induces the release of chemokines and ATP from endothelial cells (red). Phagocytes (blue) become hyperinflammatory, modify their metabolism, and produce pro-inflammatory cytokines such as IL-1β and IL-6. IL-1β can also be induced by extracellular stressors such as ATP. In this manner, the endothelial cell-phagocyte circuit sustains inflammation. During viral infections (right), oxPAPC released from infected-dead cells or from surfactant oxidation interacts with endothelial cells (red) that produce chemokines and TF. Low doses of oxPAPC (early steps of infection) elicit barrier function, while high doses of oxPAPC (late steps of infections) disrupt the endothelial barrier. Phagocytes (blue) activate inflammasome-dependent responses, secrete cytokines and TF and lead to inflammation and coagulation.

Hyperlipidemic humans and animals exhibit high levels of oxPLs, derived from oxLDLs and dead cells in their plasma and atherosclerotic plaques (133–135). These modified molecules control plaque inflammation and progression, and play a key role in the etiology of atherosclerosis (**Figure 2**). Selective oxPL neutralization, mediated by the ectopic expression of E06 antibody (136) single-chain variable fragment (E06-scFv) in high-fat fed mice that are deficient in LDL receptor (LDLR), results in severe reduction and slowing of pathology (21). In this hypercholesterolemic model, E06-scFv binds oxPLs but not unoxidized PLs, impairs pro-inflammatory macrophage activation in the aorta, and diminishes the *in locus* recruitment of monocytes and lymphocytes – this in turn reduces local and systemic inflammation. Thus, E06-scFv decreases the formation of atherosclerotic lesions and prevents valve dysfunction (21). These findings are supported by a report that quenching of reactive dicarbonyls also reduces atherosclerosis in LDLR-deficient mice (137). Indeed, oxidative reactions in the *sn-2* unsaturated chain of PLs may generate highly reactive dicarbonyl moieties such as 4-oxo-nonenal (4-ONE), malondialdehyde (MDA) and isolevuglandins (IsoLGs) (138), which covalently bind proteins and other macromolecules. Thus, use of the dicarbonyl scavenger 2-hydroxybenzylamine (2-HOBA) to block the production of molecular adducts induced by oxPL species reduces systemic inflammation and increases plaque stability (137).

Interfering with the metabolic program induced in phagocytes also indirectly dampens the pro-atherogenic effects of oxPLs. oxPAPC induces glutamine utilization, ACLY-dependent OAA accumulation, and HIF-1α stabilization, and also boosts IL-1β expression. Systemic administration of glutaminolysis or ACLY inhibitors in hypercholesterolemic mice reduces early plaque formation and decreases the production of IL-1β by macrophages in the aorta (60). Additionally, peripheral blood transcriptional signatures from Framingham Heart Study (FHS) (139) participants with pro-atherogenic lipidemia reveal an enrichment of genes that control the same metabolic pathways described for oxPAPC-treated murine macrophages (60) - this indicates that similar metabolic rearrangements are shared between humans and mice, and that metabolic intervention could be a new clinical tool for treating atherosclerosis.

IL-1β produced by myeloid cells is a crucial mediator of atherosclerosis progression (140–142): it acts systemically and in the plaque on bystander cells to augment expression of adhesion molecules and proliferation (143–146). The essential role of this cytokine in atherosclerosis and CVDs has been recently highlighted in the Canakinumab Anti-Inflammatory Thrombosis Outcomes Study (CANTOS) trial: treatment with a monoclonal antibody against IL-1β (canakinumab) proved to be protective against cardiovascular dysfunctions in patients with a history of myocardial infarction (MI) and elevated high-sensitivity C-reactive protein (CRP) (147). Single-cell transcriptome analyses of human and murine atherosclerotic lesions have mapped immune populations that participate in plaque inflammation, and underscore the major role of IL-1β (148–151). Of note, lipid-laden macrophages (described as “foamy” BODIPY^hi^SSC^hi^ or TREM2^hi^ cells) are not pro-inflammatory, while “non-foamy” CCR2^+^ macrophages are strongly enriched in inflammatory transcripts, including for IL-1β (148, 149, 151). Notably, macrophages treated with oxPAPC do not acquire a foamy phenotype and hugely upregulate IL-1β (60). Based on these reports, we speculate that the phenotype of inflammatory lesional non-foamy macrophages is driven by the metabolic program induced by oxPLs. And despite our lack of knowledge about the exact mechanisms that control the cellular and molecular dynamics induced by oxPLs in atheroma, we also propose that IL-1β release from these cells is due either to the direct action of oxPLs on macrophages (hyperactivation) or to canonical inflammasome activation. In the latter case, progressive accumulation of extracellular material such as cholesterol crystals (140) may provide the initiation signals for the activation of the NLRP3 inflammasome. In addition, macrophages and endothelial cells can form a functional circuit controlled by oxPLs (**Figure 2**). Indeed, oxPLs reportedly trigger the production of chemotactic mediators such as CCL2 and CXCL8 from endothelial cells (152–155), and recruit monocytes, thereby increasing the number of oxPL-responsive cells. oxPLs also stimulate purine release from endothelial cells and, *via* a metabolic reprograming that is controlled by mitochondrial methylenetetrahydrofolate dehydrogenase/cyclohydrolase (MTHFD2), also compensate for loss of ATP (156). The extracellular ATP released by endothelial cells can, then, activate the NLRP3 inflammasome in macrophages and trigger IL-1β secretion (89). We also posit that the nature and magnitude of inflammasome activation reflects the progression status of the atherosclerotic plaque: thus, following a dramatic increase of extracellular material in the arterial wall, a prevalence of hyperactivated macrophages is observed at early stages, and then a slow shift toward a pyroptotic phenotype takes place at later stages.

Besides production of IL-1β and other pro-inflammatory mediators, phagocytes carry out numerous functions that are dysregulated in atherosclerosis. For example, removal of dead cells is an essential anti-inflammatory process that slows down the progression of atherosclerotic lesions (157). oxPAPC alters actin polymerization in macrophages, and thereby reduces their phagocytic activity (158). oxPLs may decrease the clearance of dead cells, and thus favor inflammation and plaque widening. Lastly, long-lived inflammatory phagocytes induced by oxPLs promote and sustain the activation and proliferation of CD4^+^ T cells (56, 113), which in turn maintain chronic inflammation. This effect is further fueled by the capacity of some oxPAPC components, such as 1- palmitoyl-2-glutaroyl-sn-glycero-3-phosphorylcholine (PGPC), to enhance the ability of antigen presenting cells to migrate to the draining lymph nodes and thus potentiate T cell-dependent responses (112).

In sum, the above findings collectively establish the role of oxPLs in the induction and progression of atherosclerosis, but the proposed cellular and molecular mechanisms that underlie these effects remain to be verified.

## Lung Infections: Perspectives on a New Role of Oxidized Phospholipid in COVID19

Pulmonary surfactant forms a film at the alveolar air-liquid interface and lowers surface tension, thereby preventing atelectasis during breathing. Surfactant is a complex mixture of lipids and proteins, whose primary components (90-80%) are saturated PLs (such as 1,2-dipalmitoyl-sn-glycero-3-phosphocholine (DPPC)), which are active tension-lowering agents and are unreactive to air oxidation (7–9). Surfactant also contains (4-6%) unsaturated PLs (such as PAPC (7–9)) that can be oxidized (as discussed above). Under physiological conditions, surfactant is protected from atmospheric oxygen by antioxidant processes and by its rapid turnover. The first mechanism is mediated by specific proteins, for example surfactant protein A (SP-A) (159). The second one is carried out by type II pneumocytes and alveolar macrophages, which control the production/recycling and degradation of surfactant respectively (160–162).

Under stress, surfactant/lung homeostasis can be altered, leading to oxidation of PUFA moieties contained in pulmonary PLs. Several infections and treatments, such as acid aspiration, influenza viruses (H5N1, H1N1 and H3N2), Monkeypox virus, *Yersinia pestis*, *Bacillus anthracis* and severe acute respiratory syndrome coronavirus (SARS-CoV) (20, 163) can induce pulmonary oxPAPC accumulation, which is associated with a detrimental pro-inflammatory response, acute injury, and organ failure (20) (**Figure 2**). These detriment effects are triggered primarily by pathogen-induced generation of ROS from alveolar macrophages. Indeed, the genetic absence of NCF1 (neutrophil cytosolic factor 1) (a key component of the NADPH oxidase complex that is required for ROS production) in mice treated with H5N1 virus reduces generation of oxPL in the lung and alleviates lung dysfunctions (20). Once produced, oxPAPC modulates the inflammatory responses of macrophages, and boosts the production of cytokines such as IL-6 (20). oxPAPC also acts on endothelial cells. Although low doses of oxPAPC enhance the function of the lung endothelial barrier by remodeling the cytoskeleton and tightening cell-cell contacts (164–167), higher doses of oxPAPC, or its fragmented products, have opposite effects, disrupting endothelial barrier integrity (168, 169). This explains how pathogen-induced damage, inflammatory mediators secreted by macrophages and endothelial cell alterations can drive acute lung injury (ALI).

Coronavirus disease 2019 (COVID-19) that is caused by SARS-CoV-2 has become a global pandemic that threatens the lives of hundreds of millions of individuals around the world. SARS-CoV-2 causes mild respiratory symptoms, including fever and cough; but in some subjects it can degenerate to viral pneumonia and acute respiratory distress syndrome (ARDS). Uncontrolled pathology can lead to a cytokine storm, multi-organ failure, septic shock and coagulation abnormalities, which can lead to severe thromboembolic events (170).

SARS-CoV-2 shares 79.6% genomic sequence identity with SARS-CoV, and these two viruses likely share many features of their biology and pathogenesis (170). Notably, quantitative lipidomic and metabolomic profiling of plasma from COVID-19 patients reveals profound metabolic dysregulation, with enhanced oxidative stress and alteration of PUFA-PC homeostasis (171). These data suggest that oxPLs, which accumulate during SARS-CoV infections, also form during SARS-CoV-2 infections, and play a central role in maintaining harmful inflammatory responses. COVID-19 patients show high neutrophilia (172, 173). Since neutrophils are the major producers of ROS (174), we hypothesize that surfactant composition is extremely altered with the massive oxPAPC formation during SARS-CoV-2 infections. Moreover, high levels of IL-1β and IL-6 have been identified in SARS-CoV-2-infected subjects (175), and single-cell transcriptomic analysis of peripheral blood in COVID-19 patients also show increased subsets of IL-1β-producing monocytes (176). In addition, pulmonary arterial thrombosis has been detected in autopsy from SARS-CoV-2 patients (177, 178). In fact, all of these effects can be credited to inflammasome activation (179), which also drives the release of tissue factor (TF) (180, 181), an initiator of the coagulation cascade. Thus, oxPAPC, as an inflammasome modulator, could elicit IL-1β and TF, and coordinate inflammation as well as hemostasis during COVID-19 infection. Indeed, CD14, that regulates inflammasome activation in phagocytes in response to oxPAPC (182), as been proposed as a possible therapeutic target against COVID-19 (183). Lastly, phagocytes infected with SARS-CoV-2 remodel their metabolism and activate HIF-1α to sustain the cytokine storm (182). Accordingly, we propose that the oxPAPC that is produced during viral infections could also act on cellular metabolism, favoring ROS production – in a feed-forward loop. Although not yet validated experimentally, we propose that this detrimental loop feeds PUFA-PC oxidation and controls transcriptional responses *via* regulation of metabolite production.

## Conclusions and Future Directions

Immune cells control tissue homeostasis and respond rapidly to noxious stimuli to maintain physiological conditions. oxPLs are endogenous stressors that reprogram phagocyte metabolism and boost their pro-inflammatory responses, inducing a novel hyperinflammatory phenotype that sustains chronic inflammatory diseases. Several studies focused on oxPAPC have elucidated several molecular events that underlie its effects on phagocytes, but some questions remain unresolved: 1) Given that oxPAPC consists of a mix of biomolecules, and single oxPAPC components can have redundant or even antagonistic effects, what are the metabolic and/or inflammatory responses of unique oxPAPC species? 2) What are the receptors/targets/pathways of oxPAPC that are necessary for inducing its metabolic and/or inflammatory activities? 3) How does oxPAPC modulate the responsivity of phagocytes to other endogenous or exogenous stressors? 4) How does oxPAPC sustain cell viability when the NLR3 inflammasome is activated? 5) Does oxPAPC modulate other processes in phagocytes, such as differentiation, proliferation, motility or migration?

Since oxPLs are virtually always present during inflammation (i.e. through neutrophil-dependent ROS release or tissue damage), we anticipate that identifying their biological targets will be vital for creating new therapies against pathologies initiated by exogenous agents, such as sepsis or cytokine storm, or by endogenous moieties, such as atherosclerosis.

## Author Contributions

MG conceived and wrote the manuscript, and drew the figures. IZ was involved in discussing the contents of the paper and contributed to the writing. All authors contributed to the article and approved the submitted version.

## Funding

IZ is supported by NIH grants 1R01AI121066, 1R01DK115217, and NIAID-DAIT-NIHAI201700100. IZ holds an Investigators in the Pathogenesis of Infectious Disease Award from the Burroughs Wellcome Fund.

## Conflict of Interest

IZ reports compensation for consulting services with Implicit Biosciences.

The remaining author declares that the research was conducted in the absence of any commercial or financial relationships that could be construed as a potential conflict of interest.

## References

[1] Paolicelli RC Bolasco G Pagani F Maggi L Scianni M Panzanelli P. Synaptic Pruning by Microglia Is Necessary for Normal Brain Development. Science (2011) 333:1456–8. 10.1126/science.1202529 21778362

[2] Sinder BP Pettit AR McCauley LK. Macrophages: Their Emerging Roles in Bone. J Bone Mineral Res (2015) 30:2140–9. 10.1002/jbmr.2735 PMC4876707 26531055

[3] Hulsmans M Clauss S Xiao L Aguirre AD King KR Hanley A. Macrophages Facilitate Electrical Conduction in the Heart. Cell (2017) 169:510–22.e20. 10.1016/j.cell.2017.03.050 28431249 PMC5474950

[4] Muller PA Koscsó B Rajani GM Stevanovic K Berres M-L Hashimoto D. Crosstalk between Muscularis Macrophages and Enteric Neurons Regulates Gastrointestinal Motility. Cell (2014) 158:1210. 10.1016/j.cell.2014.08.002 28917294

[5] Xu H Barnes GT Yang Q Tan G Yang D Chou CJ. Chronic inflammation in fat plays a crucial role in the development of obesity-related insulin resistance. J Clin Invest (2003) 112:1821–30. 10.1172/JCI200319451 PMC296998 14679177

[6] Lorent JH Levental KR Ganesan L Rivera-Longsworth G Sezgin E Doktorova M. Plasma membranes are asymmetric in lipid unsaturation, packing and protein shape. Nat Chem Biol (2020) 16:644–52. 10.1038/s41589-020-0529-6 PMC7246138 32367017

[7] Bernhard W Hoffmann S Dombrowsky H Rau GA Kamlage A Kappler M. Phosphatidylcholine Molecular Species in Lung Surfactant. Am J Respir Cell Mol Biol (2001) 25:725–31. 10.1165/ajrcmb.25.6.4616 11726398

[8] Postle AD Heeley EL Wilton DC. A comparison of the molecular species compositions of mammalian lung surfactant phospholipids. Comp Biochem Physiol Part A: Mol Integr Physiol (2001) 129:65–73. 10.1016/S1095-6433(01)00306-3 11369534

[9] Bernhard W Pynn CJ Jaworski A Rau GA Hohlfeld JM Freihorst J. Mass spectrometric analysis of surfactant metabolism in human volunteers using deuteriated choline. Am J Respir Crit Care Med (2004) 170:54–8. 10.1164/rccm.200401-089OC 15044202

[10] Dashti M Kulik W Hoek F Veerman EC Peppelenbosch MP. Rezaee F. A Phospholipidomic Analysis of All Defined Human Plasma Lipoproteins. Sci Rep (2011) 1:139. 10.1038/srep00139 22355656 PMC3216620

[11] Bochkov VN Kadl A Huber J Gruber F Binder BR Leitinger N. Protective role of phospholipid oxidation products in endotoxin-induced tissue damage. Nature (2002) 419:77–81. 10.1038/nature01023 12214235

[12] Ma Z Li J Yang L Mu Y Xie W Pitt B. Inhibition of LPS- and CpG DNA-induced TNF-α response by oxidized phospholipids. Am J Physiol Lung Cell Mol Physiol (2004) 286:L808–16. 10.1152/ajplung.00220.2003 14644758

[13] Nonas S Miller I Kawkitinarong K Chatchavalvanich S Gorshkova I Bochkov VN. Oxidized Phospholipids Reduce Vascular Leak and Inflammation in Rat Model of Acute Lung Injury. Am J Respir Crit Care Med (2006) 173:1130–8. 10.1164/rccm.200511-1737OC PMC2662943 16514111

[14] Huber J Vales A Mitulovic G Blumer M Schmid R Witztum JL. Oxidized membrane vesicles and blebs from apoptotic cells contain biologically active oxidized phospholipids that induce monocyte-endothelial interactions. Arterioscler Thromb Vasc Biol (2002) 22:101–7. 10.1161/hq0102.101525 11788468

[15] Chang M-K Binder CJ Miller YI Subbanagounder G Silverman GJ Berliner JA. Apoptotic Cells with Oxidation-specific Epitopes Are Immunogenic and Proinflammatory. J Exp Med (2004) 200:1359–70. 10.1084/jem.20031763 PMC2211955 15583011

[16] Chou M-Y Fogelstrand L Hartvigsen K Hansen LF Woelkers D Shaw PX. Oxidation-specific epitopes are dominant targets of innate natural antibodies in mice and humans. J Clin Invest (2009) 119:1335–49. 10.1172/JCI36800 PMC2673862 19363291

[17] Tsiantoulas D Perkmann T Afonyushkin T Mangold A Prohaska TA Papac-Milicevic N. Circulating microparticles carry oxidation-specific epitopes and are recognized by natural IgM antibodies. J Lipid Res (2015) 56:440–8. 10.1194/jlr.P054569 PMC4306697 25525116

[18] Yang M Du Q Goswami J Varley PR Chen B Wang R. Interferon regulatory factor 1-Rab27a regulated extracellular vesicles promote liver ischemia/reperfusion injury. Hepatology (2018) 67:1056–70. 10.1002/hep.29605 PMC5826835 29059701

[19] Watson AD Leitinger N Navab M Faull KF Hörkkö S Witztum JL. Structural identification by mass spectrometry of oxidized phospholipids in minimally oxidized low density lipoprotein that induce monocyte/endothelial interactions and evidence for their presence *in vivo* . J Biol Chem (1997) 272:13597–607. 10.1074/jbc.272.21.13597 9153208

[20] Imai Y Kuba K Neely GG Yaghubian-Malhami R Perkmann T van Loo G. Identification of oxidative stress and Toll-like receptor 4 signaling as a key pathway of acute lung injury. Cell (2008) 133:235–49. 10.1016/j.cell.2008.02.043 PMC7112336 18423196

[21] Que X Hung M-Y Yeang C Gonen A Prohaska TA Sun X. Oxidized phospholipids are proinflammatory and proatherogenic in hypercholesterolaemic mice. Nature (2018) 558:301–6. 10.1038/s41586-018-0198-8 PMC6033669 29875409

[22] Sun X Seidman JS Zhao P Troutman TD Spann NJ Que X. Neutralization of Oxidized Phospholipids Ameliorates Non-alcoholic Steatohepatitis. Cell Metab (2020) 31:189–206.e8. 10.1016/j.cmet.2019.10.014 31761566 PMC7028360

[23] Meriwether D Sulaiman D Volpe C Dorfman A Grijalva V Dorreh N. Apolipoprotein A-I mimetics mitigate intestinal inflammation in COX2-dependent inflammatory bowel disease model. J Clin Invest (2019) 130:3670–85. 10.1172/JCI123700 PMC6715371 31184596

[24] Cruz D Watson AD Miller CS Montoya D Ochoa M-T Sieling PA. Host-derived oxidized phospholipids and HDL regulate innate immunity in human leprosy. J Clin Invest (2008) 118:2917–28. 10.1172/JCI34189 PMC2467381 18636118

[25] Gruber F Oskolkova O Leitner A Mildner M Mlitz V Lengauer B. Photooxidation generates biologically active phospholipids that induce heme oxygenase-1 in skin cells. J Biol Chem (2007) 282:16934–41. 10.1074/jbc.M702523200 17449870

[26] Nakanishi H Iida Y Shimizu T Taguchi R. Analysis of oxidized phosphatidylcholines as markers for oxidative stress, using multiple reaction monitoring with theoretically expanded data sets with reversed-phase liquid chromatography/tandem mass spectrometry. J Chromatogr B Analyt Technol BioMed Life Sci (2009) 877:1366–74. 10.1016/j.jchromb.2008.09.041 18964370

[27] Kanter JL Narayana S Ho PP Catz I Warren KG Sobel RA. Lipid microarrays identify key mediators of autoimmune brain inflammation. Nat Med (2006) 12:138–43. 10.1038/nm1344 16341241

[28] Qin J Goswami R Balabanov R Dawson G. Oxidized phosphatidylcholine is a marker for neuroinflammation in multiple sclerosis brain. J Neurosci Res (2007) 85:977–84. 10.1002/jnr.21206 17304573

[29] Liu B Tai Y Caceres AI Achanta S Balakrishna S Shao X. Oxidized Phospholipid OxPAPC Activates TRPA1 and Contributes to Chronic Inflammatory Pain in Mice. PLoS One (2016) 11:e0165200. 10.1371/journal.pone.0165200 27812120 PMC5094666

[30] Oehler B Kistner K Martin C Schiller J Mayer R Mohammadi M. Inflammatory pain control by blocking oxidized phospholipid-mediated TRP channel activation. Sci Rep (2017) 7:5447. 10.1038/s41598-017-05348-3 28710476 PMC5511297

[31] Yang K Han X. Lipidomics: Techniques, Applications, and Outcomes Related to Biomedical Sciences. Trends Biochem Sci (2016) 41:954–69. 10.1016/j.tibs.2016.08.010 PMC5085849 27663237

[32] Araldi E Fernández-Fuertes M Canfrán-Duque A Tang W Cline GW Madrigal-Matute J. Lanosterol Modulates TLR4-Mediated Innate Immune Responses in Macrophages. Cell Rep (2017) 19:2743–55. 10.1016/j.celrep.2017.05.093 PMC5553565 28658622

[33] Lévêque M Penna A Le Trionnaire S Belleguic C Desrues B Brinchault G. Phagocytosis depends on TRPV2-mediated calcium influx and requires TRPV2 in lipids rafts: alteration in macrophages from patients with cystic fibrosis. Sci Rep (2018) 8:4310. 10.1038/s41598-018-22558-5 29523858 PMC5844937

[34] Carroll RG Zasłona Z Galván-Peña S Koppe EL Sévin DC Angiari S. An unexpected link between fatty acid synthase and cholesterol synthesis in proinflammatory macrophage activation. J Biol Chem (2018) 293:5509–21. 10.1074/jbc.RA118.001921 PMC5900750 29463677

[35] Adachi Y Itoh K Yamada T Cerveny KL Suzuki TL Macdonald P. Coincident Phosphatidic Acid Interaction Restrains Drp1 in Mitochondrial Division. Mol Cell (2016) 63:1034–43. 10.1016/j.molcel.2016.08.013 PMC5028122 27635761

[36] Ban T Ishihara T Kohno H Saita S Ichimura A Maenaka K. Molecular basis of selective mitochondrial fusion by heterotypic action between OPA1 and cardiolipin. Nat Cell Biol (2017) 19:856–63. 10.1038/ncb3560 28628083

[37] Spann NJ Garmire LX McDonald JG Myers DS Milne SB Shibata N. Regulated Accumulation of Desmosterol Integrates Macrophage Lipid Metabolism and Inflammatory Responses. Cell (2012) 151:138–52. 10.1016/j.cell.2012.06.054 PMC3464914 23021221

[38] Hsieh W-Y Zhou QD York AG Williams KJ Scumpia PO Kronenberger EB. Toll-Like Receptors Induce Signal-Specific Reprogramming of the Macrophage Lipidome. Cell Metab (2020) 32:128–43.e5. 10.1016/j.cmet.2020.05.003 32516576 PMC7891175

[39] Wei X Song H Yin L Rizzo MG Sidhu R Covey DF. Fatty acid synthesis configures the plasma membrane for inflammation in diabetes. Nature (2016) 539:294–8. 10.1038/nature20117 PMC5671339 27806377

[40] Dennis EA Deems RA Harkewicz R Quehenberger O Brown HA Milne SB. A mouse macrophage lipidome. J Biol Chem (2010) 285:39976–85. 10.1074/jbc.M110.182915 PMC3000979 20923771

[41] Castoldi A Monteiro LB van Teijlingen Bakker N Sanin DE Rana N Corrado M. Triacylglycerol synthesis enhances macrophage inflammatory function. Nat Commun (2020) 11:4107. 10.1038/s41467-020-17881-3 32796836 PMC7427976

[42] Chen B Sun Y Niu J Jarugumilli GK Wu X. Protein Lipidation in Cell Signaling and Diseases: Function, Regulation, and Therapeutic Opportunities. Cell Chem Biol (2018) 25:817–31. 10.1016/j.chembiol.2018.05.003 PMC6054547 29861273

[43] Linder ME Deschenes RJ. Palmitoylation: policing protein stability and traffic. Nat Rev Mol Cell Biol (2007) 8:74–84. 10.1038/nrm2084 17183362

[44] Lu Y Zheng Y Coyaud É Zhang C Selvabaskaran A Yu Y. Palmitoylation of NOD1 and NOD2 is required for bacterial sensing. Science (2019) 366:460–7. 10.1126/science.aau6391 31649195

[45] McDonnell E Crown SB Fox DB Kitir B Ilkayeva OR Olsen CA. Lipids reprogram metabolism to become a major carbon source for histone acetylation. Cell Rep (2016) 17:1463–72. 10.1016/j.celrep.2016.10.012 PMC5123807 27806287

[46] Narita T Weinert BT Choudhary C. Functions and mechanisms of non-histone protein acetylation. Nat Rev Mol Cell Biol (2019) 20:156–74. 10.1038/s41580-018-0081-3 30467427

[47] He M Chiang H-H Luo H Zheng Z Qiao Q Wang L. An Acetylation Switch of the NLRP3 Inflammasome Regulates Aging-Associated Chronic Inflammation and Insulin Resistance. Cell Metab (2020) 31:580–91.e5. 10.1016/j.cmet.2020.01.009 32032542 PMC7104778

[48] Swanson KV Deng M Ting JP-Y. The NLRP3 inflammasome: molecular activation and regulation to therapeutics. Nat Rev Immunol (2019) 19:477–89. 10.1038/s41577-019-0165-0 PMC7807242 31036962

[49] Dennis EA Norris PC. Eicosanoid Storm in Infection and Inflammation. Nat Rev Immunol (2015) 15:511–23. 10.1038/nri3859 PMC4606863 26139350

[50] Wollam J Antebi A. Sterol Regulation of Metabolism, Homeostasis and Development. Annu Rev Biochem (2011) 80:885–916. 10.1146/annurev-biochem-081308-165917 21495846 PMC3918218

[51] Melo T Montero-Bullón J-F Domingues P Domingues MR. Discovery of bioactive nitrated lipids and nitro-lipid-protein adducts using mass spectrometry-based approaches. Redox Biol (2019) 23:101106. 10.1016/j.redox.2019.101106 30718106 PMC6859590

[52] Dias IHK Ferreira R Gruber F Vitorino R Rivas-Urbina A Sanchez-Quesada JL. Sulfate-based lipids: Analysis of healthy human fluids and cell extracts. Chem Phys Lipids (2019) 221:53–64. 10.1016/j.chemphyslip.2019.03.009 30910732

[53] Morrow JD Hill KE Burk RF Nammour TM Badr KF Roberts LJ. A series of prostaglandin F2-like compounds are produced *in vivo* in humans by a non-cyclooxygenase, free radical-catalyzed mechanism. PNAS (1990) 87:9383–7. 10.1073/pnas.87.23.9383 PMC55169 2123555

[54] Kadl A Sharma PR Chen W Agrawal R Meher AK Rudraiah S. Oxidized phospholipid-induced inflammation is mediated by Toll-like receptor 2. Free Radic Biol Med (2011) 51:1903–9. 10.1016/j.freeradbiomed.2011.08.026 PMC3197756 21925592

[55] Shirey KA Lai W Scott AJ Lipsky M Mistry P Pletneva LM. The TLR4 antagonist Eritoran protects mice from lethal influenza infection. Nature (2013) 497:498–502. 10.1038/nature12118 23636320 PMC3725830

[56] Zanoni I Tan Y Di Gioia M Broggi A Ruan J Shi J. An endogenous caspase-11 ligand elicits interleukin-1 release from living dendritic cells. Science (2016) 352:1232–6. 10.1126/science.aaf3036 PMC5111085 27103670

[57] Zanoni I Tan Y Di Gioia M Springstead JR Kagan JC. By Capturing Inflammatory Lipids Released from Dying Cells, the Receptor CD14 Induces Inflammasome-Dependent Phagocyte Hyperactivation. Immunity (2017) 47:697–709.e3. 10.1016/j.immuni.2017.09.010 29045901 PMC5747599

[58] Yeon SH Yang G Lee HE Lee JY. Oxidized phosphatidylcholine induces the activation of NLRP3 inflammasome in macrophages. J Leukoc Biol (2017) 101:205–15. 10.1189/jlb.3VMA1215-579RR 27256568

[59] Evavold CL Ruan J Tan Y Xia S Wu H Kagan JC. The Pore-Forming Protein Gasdermin D Regulates Interleukin-1 Secretion from Living Macrophages. Immunity (2018) 48:35–44.e6. 10.1016/j.immuni.2017.11.013 29195811 PMC5773350

[60] Di Gioia M Spreafico R Springstead JR Mendelson MM Joehanes R Levy D. Endogenous oxidized phospholipids reprogram cellular metabolism and boost hyperinflammation. Nat Immunol (2020) 21:42–53. 10.1038/s41590-019-0539-2 31768073 PMC6923570

[61] Lachmandas E Boutens L Ratter JM Hijmans A Hooiveld GJ Joosten LAB. Microbial stimulation of different Toll-like receptor signalling pathways induces diverse metabolic programmes in human monocytes. Nat Microbiol (2016) 2:1–10. 10.1038/nmicrobiol.2016.246 27991883

[62] Davies LC Rice CM Palmieri EM Taylor PR Kuhns DB McVicar DW. Peritoneal tissue-resident macrophages are metabolically poised to engage microbes using tissue-niche fuels. Nat Commun (2017) 8:2074. 10.1038/s41467-017-02092-0 29234000 PMC5727035

[63] Tannahill GM Curtis AM Adamik J Palsson-McDermott EM McGettrick AF Goel G. Succinate is an inflammatory signal that induces IL-1β through HIF-1α. Nature (2013) 496:238–42. 10.1038/nature11986 PMC4031686 23535595

[64] Jha AK Huang SC-C Sergushichev A Lampropoulou V Ivanova Y Loginicheva E. Network integration of parallel metabolic and transcriptional data reveals metabolic modules that regulate macrophage polarization. Immunity (2015) 42:419–30. 10.1016/j.immuni.2015.02.005 25786174

[65] Everts B Amiel E Huang SC-C Smith AM Chang C-H Lam WY. TLR-driven early glycolytic reprogramming *via the* kinases TBK1-IKKϵ supports the anabolic demands of dendritic cell activation. Nat Immunol (2014) 15:323–32. 10.1038/ni.2833 PMC4358322 24562310

[66] Everts B Amiel E van der Windt GJW Freitas TC Chott R Yarasheski KE. Commitment to glycolysis sustains survival of NO-producing inflammatory dendritic cells. Blood (2012) 120:1422–31. 10.1182/blood-2012-03-419747 PMC3423780 22786879

[67] Haschemi A Kosma P Gille L Evans CR Burant CF Starkl P. The Sedoheptulose Kinase CARKL Directs Macrophage Polarization through Control of Glucose Metabolism. Cell Metab (2012) 15:813–26. 10.1016/j.cmet.2012.04.023 PMC3370649 22682222

[68] Palsson-McDermott EM Curtis AM Goel G Lauterbach MAR Sheedy FJ Gleeson LE. Pyruvate kinase M2 regulates Hif-1α activity and IL-1β induction and is a critical determinant of the warburg effect in LPS-activated macrophages. Cell Metab (2015) 21:65–80. 10.1016/j.cmet.2014.12.005 25565206 PMC5198835

[69] Moon J-S Hisata S Park M-A DeNicola GM Ryter SW Nakahira K. mTORC1-Induced HK1-Dependent Glycolysis Regulates NLRP3 Inflammasome Activation. Cell Rep (2015) 12:102–15. 10.1016/j.celrep.2015.05.046 PMC4858438 26119735

[70] Millet P Vachharajani V McPhail L Yoza B McCall CE. GAPDH Binding to TNF-α mRNA Contributes to Posttranscriptional Repression in Monocytes: A Novel Mechanism of Communication between Inflammation and Metabolism. J Immunol (2016) 196:2541–51. 10.4049/jimmunol.1501345 PMC4779706 26843329

[71] Bailey JD Diotallevi M Nicol T McNeill E Shaw A Chuaiphichai S. Nitric Oxide Modulates Metabolic Remodeling in Inflammatory Macrophages through TCA Cycle Regulation and Itaconate Accumulation. Cell Rep (2019) 28:218–30.e7. 10.1016/j.celrep.2019.06.018 31269442 PMC6616861

[72] Hooftman A Angiari S Hester S Corcoran SE Runtsch MC Ling C. The Immunomodulatory Metabolite Itaconate Modifies NLRP3 and Inhibits Inflammasome Activation. Cell Metab (2020) 32(3):468–78.e7. 10.1016/j.cmet.2020.07.016 PMC7422798 32791101

[73] Serbulea V Upchurch CM Schappe MS Voigt P DeWeese DE Desai BN. Macrophage phenotype and bioenergetics are controlled by oxidized phospholipids identified in lean and obese adipose tissue. Proc Natl Acad Sci U S A (2018) 115:E6254–63. 10.1073/pnas.1800544115 PMC6142199 29891687

[74] Fong G-H Takeda K. Role and regulation of prolyl hydroxylase domain proteins. Cell Death Differentiation (2008) 15:635–41. 10.1038/cdd.2008.10 18259202

[75] Arts RJW Novakovic B ter Horst R Carvalho A Bekkering S Lachmandas E. Glutaminolysis and Fumarate Accumulation Integrate Immunometabolic and Epigenetic Programs in Trained Immunity. Cell Metab (2016) 24:807–19. 10.1016/j.cmet.2016.10.008 PMC5742541 27866838

[76] Liu P-S Wang H Li X Chao T Teav T Christen S. α-ketoglutarate orchestrates macrophage activation through metabolic and epigenetic reprogramming. Nat Immunol (2017) 18:985–94. 10.1038/ni.3796 28714978

[77] Bekkering S Arts RJW Novakovic B Kourtzelis I van der Heijden CDCC Li Y. Metabolic Induction of Trained Immunity through the Mevalonate Pathway. Cell (2018) 172:135–46.e9. 10.1016/j.cell.2017.11.025 29328908

[78] Wellen KE Hatzivassiliou G Sachdeva UM Bui TV Cross JR Thompson CB. ATP-Citrate Lyase Links Cellular Metabolism to Histone Acetylation. Science (2009) 324:1076–80. 10.1126/science.1164097 PMC2746744 19461003

[79] Covarrubias AJ Aksoylar HI Yu J Snyder NW Worth AJ Iyer SS. Akt-mTORC1 signaling regulates Acly to integrate metabolic input to control of macrophage activation. eLife (2016) 5:e11612. 10.7554/eLife.11612 26894960 PMC4769166

[80] Lauterbach MA Hanke JE Serefidou M Mangan MSJ Kolbe C-C Hess T. Toll-like Receptor Signaling Rewires Macrophage Metabolism and Promotes Histone Acetylation *via* ATP-Citrate Lyase. Immunity (2019) 51:997–1011.e7. 10.1016/j.immuni.2019.11.009 31851905

[81] Zorova LD Popkov VA Plotnikov EY Silachev DN Pevzner IB Jankauskas SS. Mitochondrial membrane potential. Analyt Biochem (2018) 552:50–9. 10.1016/j.ab.2017.07.009 PMC5792320 28711444

[82] Martínez-Reyes I Diebold LP Kong H Schieber M Huang H Hensley CT. TCA Cycle and Mitochondrial Membrane Potential Are Necessary for Diverse Biological Functions. Mol Cell (2016) 61:199–209. 10.1016/j.molcel.2015.12.002 26725009 PMC4724312

[83] Yang W Nagasawa K Münch C Xu Y Satterstrom K Jeong S. Mitochondrial Sirtuin Network Reveals Dynamic SIRT3-Dependent Deacetylation in Response to Membrane Depolarization. Cell (2016) 167:985–1000.e21. 10.1016/j.cell.2016.10.016 27881304 PMC5134900

[84] Sukumar M Liu J Mehta GU Patel SJ Roychoudhuri R Crompton JG. Mitochondrial Membrane Potential Identifies Cells with Enhanced Stemness for Cellular Therapy. Cell Metab (2016) 23:63–76. 10.1016/j.cmet.2015.11.002 26674251 PMC4747432

[85] Sanin DE Matsushita M Klein Geltink RI Grzes KM van Teijlingen Bakker N Corrado M. Mitochondrial Membrane Potential Regulates Nuclear Gene Expression in Macrophages Exposed to Prostaglandin E2. Immunity (2018) 49:1021–33.e6. 10.1016/j.immuni.2018.10.011 30566880 PMC7271981

[86] Serbulea V Upchurch CM Ahern KW Bories G Voigt P DeWeese DE. Macrophages sensing oxidized DAMPs reprogram their metabolism to support redox homeostasis and inflammation through a TLR2-Syk-ceramide dependent mechanism. Mol Metab (2017) 7:23–34. 10.1016/j.molmet.2017.11.002 29153923 PMC5784323

[87] Robbins CS Hilgendorf I Weber GF Theurl I Iwamoto Y Figueiredo J-L. Local proliferation dominates lesional macrophage accumulation in atherosclerosis. Nat Med (2013) 19:1166–72. 10.1038/nm.3258 PMC3769444 23933982

[88] Monocyte-derived alveolar macrophages drive lung fibrosis and persist in the lung over the life span. Available at: https://rupress-org.ezp-prod1.hul.harvard.edu/jem/article/214/8/2387/42522/Monocyte-derived-alveolar-macrophages-drive-lung (Accessed August 31, 2020). Rockefeller University Press. 10.1084/jem.20162152 PMC5551573 28694385

[89] Zheng D Liwinski T Elinav E. Inflammasome activation and regulation: toward a better understanding of complex mechanisms. Cell Discovery (2020) 6:1–22. 10.1038/s41421-020-0167-x 32550001 PMC7280307

[90] Groß CJ Mishra R Schneider KS Médard G Wettmarshausen J Dittlein DC. K+ Efflux-Independent NLRP3 Inflammasome Activation by Small Molecules Targeting Mitochondria. Immunity (2016) 45:761–73. 10.1016/j.immuni.2016.08.010 27692612

[91] Katsnelson MA Lozada-Soto KM Russo HM Miller BA Dubyak GR. NLRP3 inflammasome signaling is activated by low-level lysosome disruption but inhibited by extensive lysosome disruption: roles for K+ efflux and Ca2+ influx. Am J Physiol Cell Physiol (2016) 311:C83–C100. 10.1152/ajpcell.00298.2015 27170638 PMC4967136

[92] Zhong Z Liang S Sanchez-Lopez E He F Shalapour S Lin X-J. New mitochondrial DNA synthesis enables NLRP3 inflammasome activation. Nature (2018) 560:198–203. 10.1038/s41586-018-0372-z 30046112 PMC6329306

[93] Luo H Mu W-C Karki R Chiang H-H Mohrin M Shin JJ. Mitochondrial Stress-Initiated Aberrant Activation of the NLRP3 Inflammasome Regulates the Functional Deterioration of Hematopoietic Stem Cell Aging. Cell Rep (2019) 26:945–54.e4. 10.1016/j.celrep.2018.12.101 30673616 PMC6371804

[94] Dostert C Pétrilli V Van Bruggen R Steele C Mossman BT Tschopp J. Innate immune activation through Nalp3 inflammasome sensing of asbestos and silica. Science (2008) 320:674–7. 10.1126/science.1156995 PMC2396588 18403674

[95] Zhang Q Raoof M Chen Y Sumi Y Sursal T Junger W. Circulating mitochondrial DAMPs cause inflammatory responses to injury. Nature (2010) 464:104–7. 10.1038/nature08780 PMC2843437 20203610

[96] Lee G-S Subramanian N Kim AI Aksentijevich I Goldbach-Mansky R Sacks DB. The calcium-sensing receptor regulates the NLRP3 inflammasome through Ca2+ and cAMP. Nature (2012) 492:123–7. 10.1038/nature11588 PMC4175565 23143333

[97] Rossol M Pierer M Raulien N Quandt D Meusch U Rothe K. Extracellular Ca2+ is a danger signal activating the NLRP3 inflammasome through G protein-coupled calcium sensing receptors. Nat Commun (2012) 3:1329. 10.1038/ncomms2339 23271661 PMC3535422

[98] Muñoz-Planillo R Kuffa P Martínez-Colón G Smith BL Rajendiran TM Núñez G. K^+^ efflux is the common trigger of NLRP3 inflammasome activation by bacterial toxins and particulate matter. Immunity (2013) 38:1142–53. 10.1016/j.immuni.2013.05.016 PMC3730833 23809161

[99] Wolf AJ Reyes CN Liang W Becker C Shimada K Wheeler ML. Hexokinase Is an Innate Immune Receptor for the Detection of Bacterial Peptidoglycan. Cell (2016) 166:624–36. 10.1016/j.cell.2016.05.076 PMC5534359 27374331

[100] Hagar JA Powell DA Aachoui Y Ernst RK Miao EA. Cytoplasmic LPS activates caspase-11: implications in TLR4-independent endotoxic shock. Science (2013) 341:1250–3. 10.1126/science.1240988 PMC3931427 24031018

[101] Kayagaki N Wong MT Stowe IB Ramani SR Gonzalez LC Akashi-Takamura S. Noncanonical Inflammasome Activation by Intracellular LPS Independent of TLR4. Science (2013) 341:1246–9. 10.1126/science.1240248 23887873

[102] Shi J Zhao Y Wang Y Gao W Ding J Li P. Inflammatory caspases are innate immune receptors for intracellular LPS. Nature (2014) 514:187–92. 10.1038/nature13683 25119034

[103] Lee BL Stowe IB Gupta A Kornfeld OS Roose-Girma M Anderson K. Caspase-11 auto-proteolysis is crucial for noncanonical inflammasome activation. J Exp Med (2018) 215:2279–88. 10.1084/jem.20180589 PMC6122968 30135078

[104] Shi J Zhao Y Wang K Shi X Wang Y Huang H. Cleavage of GSDMD by inflammatory caspases determines pyroptotic cell death. Nature (2015) 526:660–5. 10.1038/nature15514 26375003

[105] Kayagaki N Stowe IB Lee BL O’Rourke K Anderson K Warming S. Caspase-11 cleaves gasdermin D for non-canonical inflammasome signalling. Nature (2015) 526:666–71. 10.1038/nature15541 26375259

[106] Rühl S Broz P. Caspase-11 activates a canonical NLRP3 inflammasome by promoting K(+) efflux. Eur J Immunol (2015) 45:2927–36. 10.1002/eji.201545772 26173909

[107] Podrez EA Poliakov E Shen Z Zhang R Deng Y Sun M. Identification of a novel family of oxidized phospholipids that serve as ligands for the macrophage scavenger receptor CD36. J Biol Chem (2002) 277:38503–16. 10.1074/jbc.M203318200 12105195

[108] Zanoni I Ostuni R Marek LR Barresi S Barbalat R Barton GM. CD14 controls the LPS-induced endocytosis of Toll-like receptor 4. Cell (2011) 147:868–80. 10.1016/j.cell.2011.09.051 PMC3217211 22078883

[109] Chen R Brady E McIntyre TM. Human TMEM30a Promotes Uptake of Anti-tumor and Bioactive Choline Phospholipids into Mammalian Cells. J Immunol (2011) 186:3215–25. 10.4049/jimmunol.1002710 PMC3073457 21289302

[110] Dingjan I Verboogen DR Paardekooper LM Revelo NH Sittig SP Visser LJ. Lipid peroxidation causes endosomal antigen release for cross-presentation. Sci Rep (2016) 6:22064. 10.1038/srep22064 26907999 PMC4764948

[111] Kerur N Fukuda S Banerjee D Kim Y Fu D Apicella I. cGAS drives noncanonical-inflammasome activation in age-related macular degeneration. Nat Med (2018) 24:50–61. 10.1038/nm.4450 29176737 PMC5760363

[112] Zhivaki D Borriello F Chow OA Doran B Fleming I Theisen DJ. Inflammasomes within Hyperactive Murine Dendritic Cells Stimulate Long-Lived T Cell-Mediated Anti-tumor Immunity. Cell Rep (2020) 33:108381. 10.1016/j.celrep.2020.108381 33207188 PMC7727444

[113] Koltsova EK Garcia Z Chodaczek G Landau M McArdle S Scott SR. Dynamic T cell–APC interactions sustain chronic inflammation in atherosclerosis. J Clin Invest (2012) 122:3114–26. 10.1172/JCI61758 PMC3428082 22886300

[114] Chu LH Indramohan M Ratsimandresy RA Gangopadhyay A Morris EP Monack DM. The oxidized phospholipid oxPAPC protects from septic shock by targeting the non-canonical inflammasome in macrophages. Nat Commun (2018) 9:1–16. 10.1038/s41467-018-03409-3 29520027 PMC5843631

[115] Springstead JR Gugiu BG Lee S Cha S Watson AD Berliner JA. Evidence for the importance of OxPAPC interaction with cysteines in regulating endothelial cell function. J Lipid Res (2012) 53:1304–15. 10.1194/jlr.M025320 PMC3371242 22550136

[116] Muri J Feng Q Wolleb H Shamshiev A Ebner C Tortola L. Cyclopentenone Prostaglandins and Structurally Related Oxidized Lipid Species Instigate and Share Distinct Pro- and Anti-inflammatory Pathways. Cell Rep (2020) 30:4399–417.e7. 10.1016/j.celrep.2020.03.019 32234476

[117] UniProt: a worldwide hub of protein knowledge. Nucleic Acids Res (2019) 47:D506–15. 10.1093/nar/gky1049 PMC6323992 30395287

[118] Kar NS Ashraf MZ Valiyaveettil M Podrez EA. Mapping and Characterization of the Binding Site for Specific Oxidized Phospholipids and Oxidized Low Density Lipoprotein of Scavenger Receptor CD36. J Biol Chem (2008) 283:8765–71. 10.1074/jbc.M709195200 PMC2417175 18245080

[119] Gao D Ashraf MZ Kar NS Lin D Sayre LM Podrez EA. Structural Basis for the Recognition of Oxidized Phospholipids in Oxidized Low Density Lipoproteins by Class B Scavenger Receptors CD36 and SR-BI. J Biol Chem (2010) 285:4447–54. 10.1074/jbc.M109.082800 PMC2836050 19996318

[120] Lamping N Hoess A Yu B Park TC Kirschning CJ Pfeil D. Effects of site-directed mutagenesis of basic residues (Arg 94, Lys 95, Lys 99) of lipopolysaccharide (LPS)-binding protein on binding and transfer of LPS and subsequent immune cell activation. J Immunol (1996) 157:4648–56. 8906845

[121] Rühl S Shkarina K Demarco B Heilig R Santos JC Broz P. ESCRT-dependent membrane repair negatively regulates pyroptosis downstream of GSDMD activation. Science (2018) 362:956–60. 10.1126/science.aar7607 30467171

[122] Stemmer U Ramprecht C Zenzmaier E Stojčić B Rechberger G Kollroser M. Uptake and protein targeting of fluorescent oxidized phospholipids in cultured RAW 264.7 macrophages. Biochim Biophys Acta (BBA) - Mol Cell Biol Lipids (2012) 1821:706–18. 10.1016/j.bbalip.2012.01.014 PMC3790972 22333180

[123] Rogers C Erkes DA Nardone A Aplin AE Fernandes-Alnemri T Alnemri ES. Gasdermin pores permeabilize mitochondria to augment caspase-3 activation during apoptosis and inflammasome activation. Nat Commun (2019) 10:1689. 10.1038/s41467-019-09397-2 30976076 PMC6459836

[124] Humphries F Shmuel-Galia L Ketelut-Carneiro N Li S Wang B Nemmara VV. Succination inactivates gasdermin D and blocks pyroptosis. Science (2020) 369 (6511):1633–7. 10.1126/science.abb9818 PMC8744141 32820063

[125] Hu JJ Liu X Xia S Zhang Z Zhang Y Zhao J. FDA-approved disulfiram inhibits pyroptosis by blocking gasdermin D pore formation. Nat Immunol (2020) 21:736–45. 10.1038/s41590-020-0669-6 PMC7316630 32367036

[126] Gaidt MM Ebert TS Chauhan D Schmidt T Schmid-Burgk JL Rapino F. Human Monocytes Engage an Alternative Inflammasome Pathway. Immunity (2016) 44:833–46. 10.1016/j.immuni.2016.01.012 27037191

[127] Libby P Buring JE Badimon L Hansson GK Deanfield J Bittencourt MS. Atherosclerosis. Nat Rev Dis Primers (2019) 5:1–18. 10.1038/s41572-019-0106-z 31420554

[128] Geng S Chen K Yuan R Peng L Maitra U Diao N. The persistence of low-grade inflammatory monocytes contributes to aggravated atherosclerosis. Nat Commun (2016) 7:1–15. 10.1038/ncomms13436 PMC5105176 27824038

[129] Carnevale R Nocella C Petrozza V Cammisotto V Pacini L Sorrentino V. Localization of lipopolysaccharide from Escherichia Coli into human atherosclerotic plaque. Sci Rep (2018) 8:3598. 10.1038/s41598-018-22076-4 29483584 PMC5826929

[130] Williams JW Zaitsev K Kim K-W Ivanov S Saunders BT Schrank PR. Limited proliferation capacity of aortic intima resident macrophages requires monocyte recruitment for atherosclerotic plaque progression. Nat Immunol (2020) 21:1194–204. 10.1038/s41590-020-0768-4 PMC7502558 32895539

[131] Wang Y Dubland Joshua A Allahverdian S Asonye E Sahin B Jaw JE. Smooth Muscle Cells Contribute the Majority of Foam Cells in ApoE (Apolipoprotein E)-Deficient Mouse Atherosclerosis. Arteriosclerosis Thrombosis Vasc Biol (2019) 39:876–87. 10.1161/ATVBAHA.119.312434 PMC6482082 30786740

[132] Ruiz JL Weinbaum S Aikawa E Hutcheson JD. Zooming in on the genesis of atherosclerotic plaque microcalcifications. J Physiol (Lond) (2016) 594:2915–27. 10.1113/JP271339 PMC4887667 27040360

[133] Frey B Haupt R Alms S Holzmann G König T Kern H. Increase in fragmented phosphatidylcholine in blood plasma by oxidative stress. J Lipid Res (2000) 41:1145–53. 10.1016/S0022-2275(20)32021-6 10884297

[134] Podrez EA Byzova TV Febbraio M Salomon RG Ma Y Valiyaveettil M. Platelet CD36 links hyperlipidemia, oxidant stress and a prothrombotic phenotype. Nat Med (2007) 13:1086–95. 10.1038/nm1626 PMC3042888 17721545

[135] Oskolkova OV Afonyushkin T Preinerstorfer B Bicker W Schlieffen Ev Hainzl E. Oxidized Phospholipids Are More Potent Antagonists of Lipopolysaccharide than Inducers of Inflammation. J Immunol (2010) 185:7706–12. 10.4049/jimmunol.0903594 21068406

[136] Palinski W Hörkkö S Miller E Steinbrecher UP Powell HC Curtiss LK. Cloning of monoclonal autoantibodies to epitopes of oxidized lipoproteins from apolipoprotein E-deficient mice. Demonstration of epitopes of oxidized low density lipoprotein in human plasma. J Clin Invest (1996) 98:800–14. 10.1172/JCI118853 PMC507491 8698873

[137] Tao H Huang J Yancey PG Yermalitsky V Blakemore JL Zhang Y. Scavenging of reactive dicarbonyls with 2-hydroxybenzylamine reduces atherosclerosis in hypercholesterolemic Ldlr –/– mice. Nat Commun (2020) 11:4084. 10.1038/s41467-020-17915-w 32796843 PMC7429830

[138] Bochkov VN Oskolkova OV Birukov KG Levonen A-L Binder CJ Stöckl J. Generation and Biological Activities of Oxidized Phospholipids. Antioxid Redox Signal (2010) 12:1009–59. 10.1089/ars.2009.2597 PMC3121779 19686040

[139] Mahmood SS Levy D Vasan RS Wang TJ. The Framingham Heart Study and the epidemiology of cardiovascular disease: a historical perspective. Lancet (2014) 383:999–1008. 10.1016/S0140-6736(13)61752-3 24084292 PMC4159698

[140] Duewell P Kono H Rayner KJ Sirois CM Vladimer G Bauernfeind FG. NLRP3 inflammasomes are required for atherogenesis and activated by cholesterol crystals. Nature (2010) 464:1357–61. 10.1038/nature08938 PMC2946640 20428172

[141] Hendrikx T Jeurissen MLJ van Gorp PJ Gijbels MJ Walenbergh SMA Houben T. Bone marrow-specific caspase-1/11 deficiency inhibits atherosclerosis development in Ldlr(-/-) mice. FEBS J (2015) 282:2327–38. 10.1111/febs.13279 25817537

[142] Fuster JJ MacLauchlan S Zuriaga MA Polackal MN Ostriker AC Chakraborty R. Clonal hematopoiesis associated with TET2 deficiency accelerates atherosclerosis development in mice. Science (2017) 355:842–7. 10.1126/science.aag1381 PMC5542057 28104796

[143] Bevilacqua MP Pober JS Majeau GR Cotran RS Gimbrone MA. Interleukin 1 (IL-1) induces biosynthesis and cell surface expression of procoagulant activity in human vascular endothelial cells. J Exp Med (1984) 160:618–23. 10.1084/jem.160.2.618 PMC2187463 6332168

[144] Bevilacqua MP Pober JS Wheeler ME Cotran RS Gimbrone MA. Interleukin 1 acts on cultured human vascular endothelium to increase the adhesion of polymorphonuclear leukocytes, monocytes, and related leukocyte cell lines. J Clin Invest (1985) 76:2003–11. 10.1172/JCI112200 PMC424265 3877078

[145] Libby P Warner SJ Friedman GB. Interleukin 1: a mitogen for human vascular smooth muscle cells that induces the release of growth-inhibitory prostanoids. J Clin Invest (1988) 81:487–98. 10.1172/JCI113346 PMC329596 3276731

[146] Dinarello CA. Immunological and Inflammatory Functions of the Interleukin-1 Family. Annu Rev Immunol (2009) 27:519–50. 10.1146/annurev.immunol.021908.132612 19302047

[147] Ridker PM Everett BM Thuren T MacFadyen JG Chang WH Ballantyne C. Antiinflammatory Therapy with Canakinumab for Atherosclerotic Disease. N Engl J Med (2017) 377:1119–31. 10.1056/NEJMoa1707914 28845751

[148] Kim K Shim D Lee JS Zaitsev K Williams JW Kim K-W. Transcriptome Analysis Reveals Non-Foamy Rather than Foamy Plaque Macrophages Are Pro-Inflammatory in Atherosclerotic Murine Models. Circ Res (2018) 123:1127–42. 10.1161/CIRCRESAHA.118.312804 PMC6945121 30359200

[149] Cochain C Vafadarnejad E Arampatzi P Pelisek J Winkels H Ley K. Single-Cell RNA-Seq Reveals the Transcriptional Landscape and Heterogeneity of Aortic Macrophages in Murine Atherosclerosis. Circ Res (2018) 122:1661–74. 10.1161/CIRCRESAHA.117.312509 29545365

[150] Fernandez DM Rahman AH Fernandez NF Chudnovskiy A Amir ED Amadori L. Single-cell immune landscape of human atherosclerotic plaques. Nat Med (2019) 25:1576–88. 10.1038/s41591-019-0590-4 PMC7318784 31591603

[151] Zernecke A Winkels H Cochain C Williams Jesse W Wolf D Soehnlein O. Meta-Analysis of Leukocyte Diversity in Atherosclerotic Mouse Aortas. Circ Res (2020) 127:402–26. 10.1161/CIRCRESAHA.120.316903 PMC7371244 32673538

[152] Leitinger N Tyner TR Oslund L Rizza C Subbanagounder G Lee H. Structurally similar oxidized phospholipids differentially regulate endothelial binding of monocytes and neutrophils. Proc Natl Acad Sci USA (1999) 96:12010–5. 10.1073/pnas.96.21.12010 PMC18403 10518567

[153] Shih PT Elices MJ Fang ZT Ugarova TP Strahl D Territo MC. Minimally modified low-density lipoprotein induces monocyte adhesion to endothelial connecting segment-1 by activating beta1 integrin. J Clin Invest (1999) 103:613–25. 10.1172/JCI5710 PMC479707 10074478

[154] Lee H Shi W Tontonoz P Wang S Subbanagounder G Hedrick CC. Role for peroxisome proliferator-activated receptor alpha in oxidized phospholipid-induced synthesis of monocyte chemotactic protein-1 and interleukin-8 by endothelial cells. Circ Res (2000) 87:516–21. 10.1161/01.res.87.6.516 10988245

[155] Subbanagounder G Wong JW Lee H Faull KF Miller E Witztum JL. Epoxyisoprostane and epoxycyclopentenone phospholipids regulate monocyte chemotactic protein-1 and interleukin-8 synthesis. Formation of these oxidized phospholipids in response to interleukin-1beta. J Biol Chem (2002) 277:7271–81. 10.1074/jbc.M107602200 11751881

[156] Pober JS Sessa WC. Evolving functions of endothelial cells in inflammation. Nat Rev Immunol (2007) 7:803–15. 10.1038/nri2171 17893694

[157] Yurdagul A Doran AC Cai B Fredman G Tabas IA. Mechanisms and Consequences of Defective Efferocytosis in Atherosclerosis. Front Cardiovasc Med (2018) 4:86. 10.3389/fcvm.2017.00086 29379788 PMC5770804

[158] Matt U Sharif O Martins R Furtner T Langeberg L Gawish R. WAVE1 mediates suppression of phagocytosis by phospholipid-derived DAMPs. J Clin Invest (2013) 123:3014–24. 10.1172/JCI60681 PMC3696563 23934128

[159] Kuzmenko AI Wu H Bridges JP McCormack FX. Surfactant lipid peroxidation damages surfactant protein A and inhibits interactions with phospholipid vesicles. J Lipid Res (2004) 45:1061–8. 10.1194/jlr.M300360-JLR200 15026426

[160] Yoshida M Ikegami M Reed JA Chroneos ZC Whitsett JA. GM-CSF regulates protein and lipid catabolism by alveolar macrophages. Am J Physiol Lung Cell Mol Physiol (2001) 280:L379–86. 10.1152/ajplung.2001.280.3.L379 11159019

[161] Baker AD Malur A Barna BP Ghosh S Kavuru MS Malur AG. Targeted PPARγ deficiency in alveolar macrophages disrupts surfactant catabolism. J Lipid Res (2010) 51:1325–31. 10.1194/jlr.M001651 PMC3035495 20064973

[162] Fukuzawa T Ishida J Kato A Ichinose T Ariestanti DM Takahashi T. Lung Surfactant Levels are Regulated by Ig-Hepta/GPR116 by Monitoring Surfactant Protein D. PloS One (2013) 8:e69451. 10.1371/journal.pone.0069451 23922714 PMC3726689

[163] Lee Y-H Lai C-L Lai C-L Hsieh S-H Shieh C-C Huang L-M. Influenza A virus induction of oxidative stress and MMP-9 is associated with severe lung pathology in a mouse model. Virus Res (2013) 178:411–22. 10.1016/j.virusres.2013.09.011 24055463

[164] Birukov Konstantin G Bochkov Valery N Birukova Anna A Kawkitinarong K Rios A Leitner A. Epoxycyclopentenone-Containing Oxidized Phospholipids Restore Endothelial Barrier Function *via* Cdc42 and Rac. Circ Res (2004) 95:892–901. 10.1161/01.RES.0000147310.18962.06 15472119

[165] Singleton PA Chatchavalvanich S Fu P Xing J Birukova AA Fortune JA. Akt-Mediated Transactivation of the S1P1 Receptor in Caveolin-Enriched Microdomains Regulates Endothelial Barrier Enhancement by Oxidized Phospholipids. Circ Res (2009) 104:978–86. 10.1161/CIRCRESAHA.108.193367 PMC3163385 19286607

[166] Birukova AA Zebda N Cokic I Fu P Wu T Dubrovskyi O. p190RhoGAP mediates protective effects of oxidized phospholipids in the models of ventilator induced lung injury. Exp Cell Res (2011) 317:859–72. 10.1016/j.yexcr.2010.11.011 PMC3057230 21111731

[167] Birukova AA Singleton PA Gawlak G Tian X Mirzapoiazova T Mambetsariev B. GRP78 is a novel receptor initiating a vascular barrier protective response to oxidized phospholipids. Mol Biol Cell (2014) 25:2006–16. 10.1091/mbc.E13-12-0743 PMC4072574 24829380

[168] Starosta V Wu T Zimman A Pham D Tian X Oskolkova O. Differential regulation of endothelial cell permeability by high and low doses of oxidized 1-palmitoyl-2-arachidonyl-sn-glycero-3-phosphocholine. Am J Respir Cell Mol Biol (2012) 46:331–41. 10.1165/rcmb.2011-0153OC PMC3326435 21997484

[169] Birukova AA Starosta V Tian X Higginbotham K Koroniak L Berliner JA. Fragmented oxidation products define barrier disruptive endothelial cell response to OxPAPC. Transl Res (2013) 161:495–504. 10.1016/j.trsl.2012.12.008 23305708 PMC3660521

[170] Nishiga M Wang DW Han Y Lewis DB Wu JC. COVID-19 and cardiovascular disease: from basic mechanisms to clinical perspectives. Nat Rev Cardiol (2020) 17:543–58. 10.1038/s41569-020-0413-9 PMC7370876 32690910

[171] Song J-W Lam SM Fan X Cao W-J Wang S-Y Tian H. Omics-Driven Systems Interrogation of Metabolic Dysregulation in COVID-19 Pathogenesis. Cell Metab (2020) 32:188–202.e5. 10.1016/j.cmet.2020.06.016 32610096 PMC7311890

[172] Morrissey SM Geller AE Hu X Tieri D Cooke EA Ding C. Emergence of Low-density Inflammatory Neutrophils Correlates with Hypercoagulable State and Disease Severity in COVID-19 Patients. medRxiv (2020). 10.1101/2020.05.22.20106724

[173] Leppkes M Knopf J Naschberger E Lindemann A Singh J Herrmann I. Vascular occlusion by neutrophil extracellular traps in COVID-19. EBioMedicine (2020) 58:2352–3964. 10.1016/j.ebiom.2020.102925 PMC7397705 32745993

[174] Nguyen GT Green ER Mecsas J. Neutrophils to the ROScue: Mechanisms of NADPH Oxidase Activation and Bacterial Resistance. Front Cell Infect Microbiol (2017) 7:373. 10.3389/fcimb.2017.00373 28890882 PMC5574878

[175] He L Ding Y Zhang Q Che X He Y Shen H. Expression of elevated levels of pro-inflammatory cytokines in SARS-CoV-infected ACE2+ cells in SARS patients: relation to the acute lung injury and pathogenesis of SARS. J Pathol (2006) 210:288–97. 10.1002/path.2067 PMC7167655 17031779

[176] Wen W Su W Tang H Le W Zhang X Zheng Y. Immune cell profiling of COVID-19 patients in the recovery stage by single-cell sequencing. Cell Discovery (2020) 6:1–18. 10.1038/s41421-020-0168-9 32377375 PMC7197635

[177] Lax SF Skok K Zechner P Kessler HH Kaufmann N Koelblinger C. Pulmonary Arterial Thrombosis in COVID-19 With Fatal Outcome: Results From a Prospective, Single-Center, Clinicopathologic Case Series. Ann Intern Med (2020) 173(5):350–61. 10.7326/M20-2566 PMC7249507 32422076

[178] Wichmann D Sperhake J-P Lütgehetmann M Steurer S Edler C Heinemann A. Autopsy Findings and Venous Thromboembolism in Patients With COVID-19. Ann Intern Med (2020) 173(4):268–77. 10.7326/M20-2003 PMC7240772 32374815

[179] Rodrigues TS de Sá KSG Ishimoto AY Becerra A Oliveira S Almeida L. Inflammasomes are activated in response to SARS-CoV-2 infection and are associated with COVID-19 severity in patients. J Exp Med (2021) 218(3):e20201707. 10.1084/jem.20201707 33231615 PMC7684031

[180] Wu C Lu W Zhang Y Zhang G Shi X Hisada Y. Inflammasome Activation Triggers Blood Clotting and Host Death through Pyroptosis. Immunity (2019) 50:1401–11.e4. 10.1016/j.immuni.2019.04.003 31076358 PMC6791531

[181] Yang X Cheng X Tang Y Qiu X Wang Y Kang H. Bacterial Endotoxin Activates the Coagulation Cascade through Gasdermin D-Dependent Phosphatidylserine Exposure. Immunity (2019) 51:983–96.e6. 10.1016/j.immuni.2019.11.005 31836429

[182] Di Gioia M Zanoni I. CD14: Not Just Chaperone, But a Key-Player in Inflammation. In: Rossetti C Peri F (eds) The Role of Toll-Like Receptor 4 in Infectious and Non Infectious Inflammation. Progress in Inflammation Research. Springer, Cham (2021) vol 87. p 57–78. 10.1007/978-3-030-56319-6_4

[183] Martin TR Wurfel MM Zanoni I Ulevitch R. Targeting innate immunity by blocking CD14: Novel approach to control inflammation and organ dysfunction in COVID-19 illness. EBioMedicine (2020) 57:102836. 10.1016/j.ebiom.2020.102836 32574958 PMC7305752

[184] Codo AC Davanzo GG Monteiro L de B de Souza GF Muraro SP Virgilio-da-Silva JV. Elevated Glucose Levels Favor SARS-CoV-2 Infection and Monocyte Response through a HIF-1α/Glycolysis-Dependent Axis. Cell Metab (2020) 32:437–46.e5. 10.1016/j.cmet.2020.07.007 32697943 PMC7367032

